# Copolymerization of Carbon Dioxide with 1,2-Butylene Oxide and Terpolymerization with Various Epoxides for Tailorable Properties

**DOI:** 10.3390/polym15030748

**Published:** 2023-02-01

**Authors:** Shuo Tang, Hongyi Suo, Rui Qu, Hao Tang, Miao Sun, Yusheng Qin

**Affiliations:** College of Chemistry and Chemical Engineering, Yantai University, Yantai 264005, China

**Keywords:** CO_2_-based polycarbonate, terpolymerization, 1,2-butylene oxide, biodegradable

## Abstract

The copolymerization of carbon dioxide (CO_2_) with epoxides demonstrates promise as a new synthetic method for low-carbon polymer materials, such as aliphatic polycarbonate materials. In this study, a binary Schiff base cobalt system was successfully used to catalyze the copolymerization of 1,2-butylene oxide (BO) and CO_2_ and its terpolymerization with other epoxides such as propylene oxide (PO) and cyclohexene oxide (CHO). ^1^H nuclear magnetic resonance (^1^H NMR), diffusion-ordered spectroscopy (DOSY), gel permeation chromatography (GPC), and differential scanning calorimetry (DSC) confirmed the successful synthesis of the alternating terpolymer. In addition, the effects of the polymerization reaction conditions and copolymerization monomer composition on the polymer structure and properties were examined systematically. By regulating the epoxide feed ratio, polycarbonates with an adjustable glass transition temperature (T_g_) (11.2–67.8 °C) and hydrophilicity (water contact angle: 85.2–95.2°) were prepared. Thus, this ternary polymerization method provides an effective method of modulating the surface hydrophobicity of CO_2_-based polymers and their biodegradation properties.

## 1. Introduction

CO_2_ is an abundant, non-toxic, and non-flammable source of waste gases [1,2,3,4]. One of the most promising pathways for CO_2_ utilization involves the use of epoxides and CO_2_ to prepare new aliphatic polycarbonate materials, which not only uses CO_2_ as a carbon resource, but also provides a potentially sustainable method to produce valuable carbon-neutral polycarbonate materials [5,6,7,8].

Previously, monomers such as propylene oxide (PO) and cyclohexene oxide (CHO) have been used most frequently for polymerization to produce aliphatic polycarbonates [9,10]. Poly(propylene carbonate) (PPC), a copolymer of CO_2_ and PO, is the most promising species for industrialization because of its good biodegradation properties. However, previous studies have shown that it is difficult to use PPC alone as a film material due to its amorphous nature and low glass transition temperature of ~35 °C, making it extremely brittle at low temperatures, as well as its poor dimensional stability at high temperatures [11]. CHO with CO_2_ can produce poly(cyclohexane carbonate) (PCHC) with a T_g_ of 115 °C [12]. However, the mechanical fragility of PCHC severely limits its practical applications. Commonly, the addition of a third monomer for terpolymerization decreases the brittleness of PCHC [12,13,14]. In addition, changes in thermal properties can also be achieved by combining different monomers into random copolymers. Typically, random copolymers have only one glass transition temperature, which lies between the T_g_ values of each homopolymer and is determined by the concentration of each component. Recently, Darensbourg and his colleagues have demonstrated an efficient one-pot strategy to synthesize CO_2_-based triblock copolymers that can be 3D-printed via direct ink writing techniques to produce porous structures that can be modified or crosslinked. ABA-type triblock copolymers were synthesized using 1,2-butylene oxide (BO) as a low T_g_ block and vinyl cyclohexene oxide (VCHO) as a high T_g_ block, yielding high thermal stability, tunable thermal transition, and good mechanical properties [15].

Similar to PO and CHO, BO is another important epoxy monomer that is widely used in industry, and the polyether prepared by its ring-opening homopolymerization exhibits hydrophobicity and a low T_g_, demonstrating good potential in polyurethane and lubrication applications. However, traditional BO manufacturing involves the chlorohydrin method, which consumes a large amount of chlorine gas and generates a great deal of waste water and residue containing organic chlorides, causing severe pollution. With the improvement of epoxidation technology in recent years, the unique process route of BO generation by 1-butene catalytic epoxidation (CHP or HPPO method) has become a popular study issue [16]. The route adopts 1-butene as a raw material, cumyl hydroperoxide or hydrogen peroxide as an oxidant, and direct epoxidation under the action of a silica–titanium molecular sieve catalyst to generate BO. Unlike the chlorohydrin technique, the CHP or HPPO route does not use chlorine gas, which avoids environmental pollution and provides the possibility of the green and low-cost production of BO. Its growth is also fostered in the new materials industry. BO-derived polyether materials have attracted considerable interest due to their unique features [17,18,19,20,21,22]. Coates et al. investigated the ring-opening copolymerization of a series of epoxides and cyclic anhydrides using [^F^Salph]AlCl and bis(triphenylphosphine)iminium chloride (PPNCl) as the catalysts [23]. The T_g_ values of the polyesters obtained from BO and the six cyclic anhydrides were less than those of the others, indicating that the introduction of methyl substituents can regulate the T_g_ of polyesters with firmly coupled ortho ester groups [24].

**Scheme 1:**
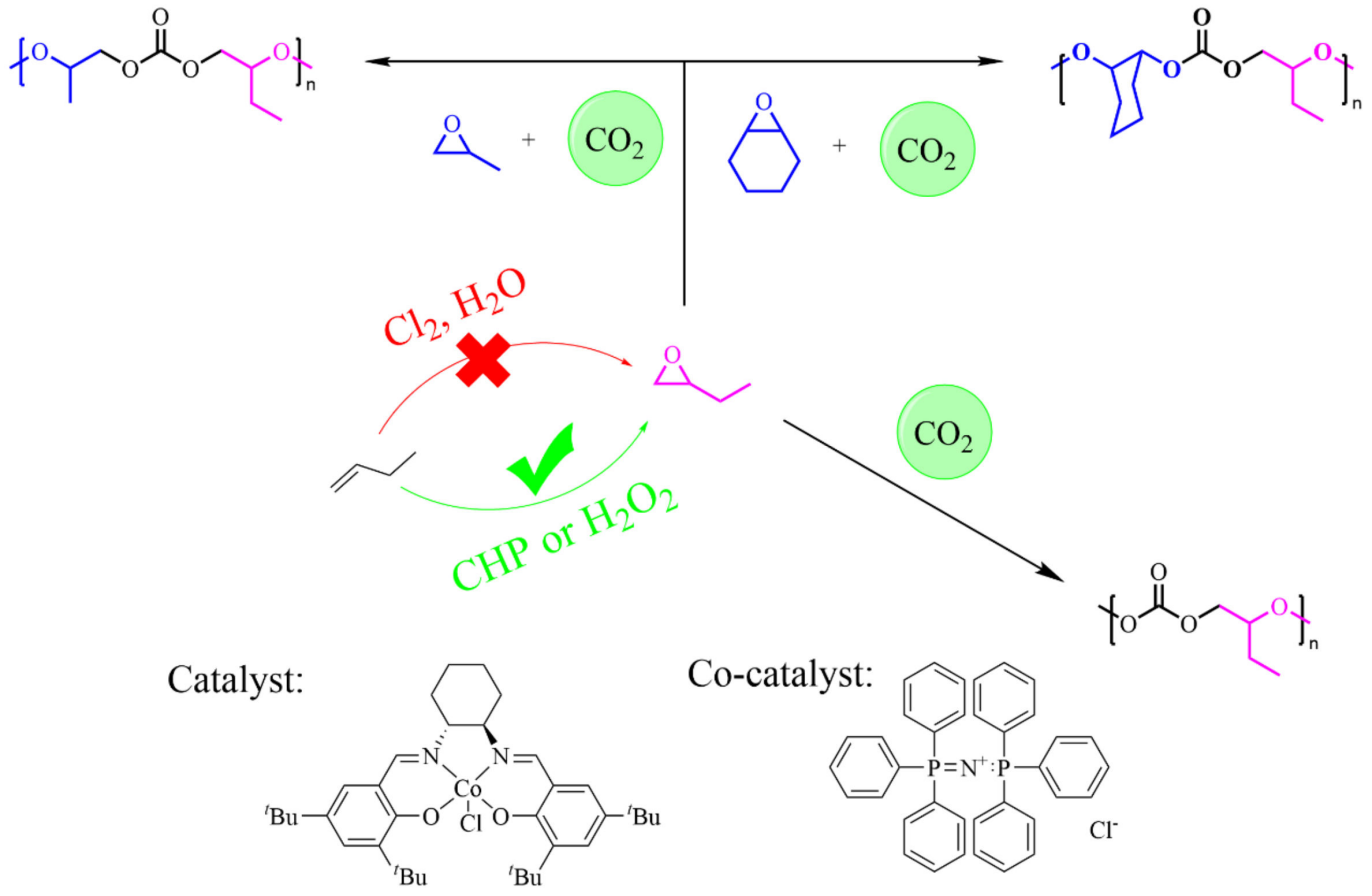
Copolymerization of CO_2_ and epoxides (BO, PO, and CHO).

Previous studies have investigated the copolymerization of BO with CO_2_ for the preparation of polycarbonate. However, most of these studies have focused on the use of BO as a reaction substrate to verify the versatility of its catalyst [25,26,27]. Systematic studies on the copolymerization of BO with CO_2_ and its polymer properties are still lacking. Considering the positive effect of BO introduction on the thermal, mechanical, and biodegradation properties of the CO_2_-based polycarbonate, it is important to investigate the copolymerization of CO_2_ with BO. However, the copolymerization of BO with CO_2_ to obtain high-molecular-weight polycarbonate is still a challenge [28,29]. A suitable, efficient catalyst system is essential to achieve high-molecular-weight polycarbonates by the copolymerization of BO and CO_2_. Various catalyst systems, including metalloporphyrins [30], zinc glutarate [31], double metal cyanide (DMC) [32], Schiff base complexes [33], and organoboron [34,35], have been used to catalyze the copolymerization of epoxides and CO_2_. Among them, the Schiff base cobalt/co-catalyst system is widely used because of its high activity. In this study, (R,R)-SalenCoCl and bis(triphenylphosphine)ammonium chloride (PPNCl) were selected as the catalyst and co-catalyst, respectively, to catalyze the copolymerization of CO_2_ with BO to prepare high-molecular-weight poly(butylene carbonate) (PBC). In addition, terpolymerization reactions of BO/PO/CO_2_ and BO/CHO/CO_2_ involving BO were investigated (Scheme 1), and the effects of structural changes on the glass transition temperature, thermal stability, and surface characteristics of polycarbonates were also examined.

## 2. Materials and Methods

### 2.1. Materials

All water- and oxygen-sensitive compounds were handled in an argon-protected glove box. PO (98%), CHO (98%), and 1,2-butylene oxide (BO, 98%) were purchased from Energy Chemical (Shanghai, China) or Aldrich (Milwaukee, USA) and used under argon after refluxing with calcium hydride. Dichloromethane (DCM), ethanol, and high-performance liquid chromatography-grade tetrahydrofuran (THF) were purchased from Energy Chemical (Shanghai, China) and used as received. Bis(triphenylphosphine)iminium chloride (PPNCl, 98%) was purchased from Energy Chemical (Shanghai, China) and used after drying under a vacuum. Carbon dioxide (99.999%) was purchased from Yantai Feiyuan Special Gases Co., Ltd (Yantai, Shandong, China). and used as received. SalenCo(III)Cl was prepared according to the method reported by Jacobsen et al. [36].

### 2.2. Characterization

All crude products were sampled into 5-mm NMR tubes and dissolved in CDCl_3_. ^1^H NMR and diffusion-ordered spectroscopy (DOSY) were recorded on a JEOL-400YH (Tokyo, Japan) instrument at a frequency of 400 MHz. The number-average relative molecular masses and relative molecular mass distributions were determined on a Waters410 gel permeation chromatography (GPC) instrument (Milford, MA, USA), using tetrahydrofuran and polystyrene as the eluent and standard, respectively. Differential scanning calorimetry measurements (DSC) of the polymer samples were conducted on a NETZSCH DSC 200F3 instrument (Selb, Germany). Samples were prepared in aluminum pans. All samples were analyzed using the following temperature program: −50 to 150 °C at 10 °C min^−1^, 150 to −50 °C at 10 °C min^−1^, and then −50 to 150 °C at 10 °C min^−1^. Data were processed using the StarE software. All of the reported T_g_ values were observed at the second heating cycle. The thermal decomposition temperature (T_d_) of the polymers was determined on a NETZSCH STA 449 F5 Jupiter system (Selb, Germany) under nitrogen. For contact angle tests, the slides were washed in acetone for 2 h by sonication, and were then placed in a solution of the polymer in DCM (5 mg mL^−1^) and incubated overnight at room temperature. After the solvent was evaporated under air, the slides were washed with deionized water to remove unadhered particles. To estimate the contact angle, the fixation drop method was used. A drop of deionized water was added to the surface for measuring the angle between the solid and liquid phases. All samples were measured six times to reduce systematic errors.

### 2.3. Copolymerization Procedure

In a glove box, the required catalyst and epoxide monomers (viz. BO, PO, or CHO) were charged into a pre-dried 10 mL autoclave equipped with a magnetic stir bar. Next, the autoclave was removed from the glove box, pressurized with CO_2_ at the preset required temperature for the required time. Polymerization was terminated by cooling the autoclave to room temperature. After releasing CO_2_, a small aliquot of the copolymerization mixture was removed for ^1^H NMR spectroscopy. The remaining crude mixture was dissolved in CH_2_Cl_2_ and then precipitated in ethanol three times to remove the residual epoxide and cyclic carbonate, affording a purple solid product. The produced polymer was used for further characterization without separating the catalyst.

## 3. Results and Discussion

### 3.1. Copolymerization of BO/CO_2_

**Figure 1:**
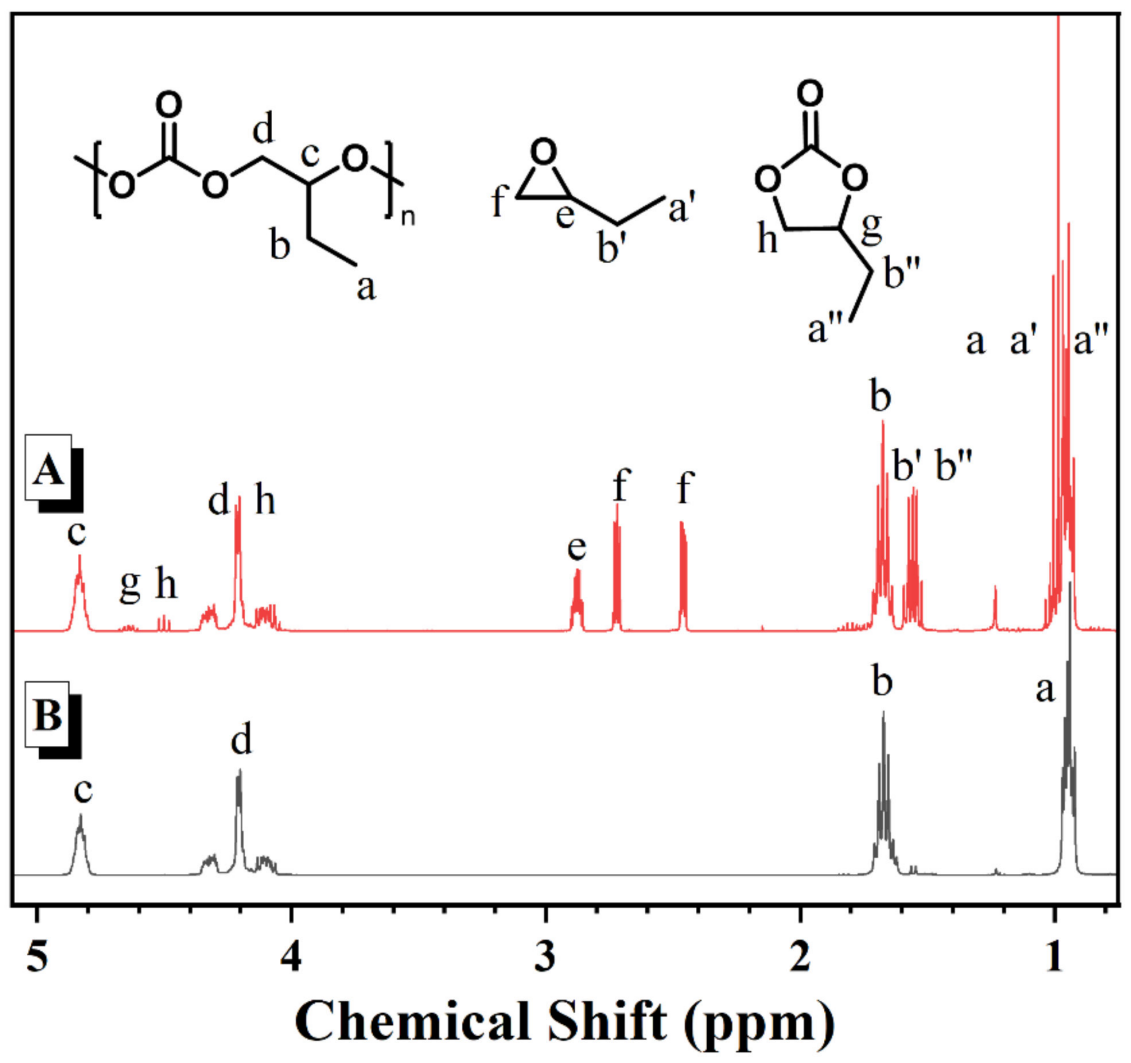
^1^H NMR spectra of crude products (**A**) and purified products (**B**) of PBC (Table 1, entry 3).

The copolymerization of CO_2_ and BO was successfully conducted by using an appropriate reaction temperature and an appropriate ratio of BO/cat/PPNCl, and the copolymerized product exhibited excellent reactivity and selectivity (Table 1). For example, PBC was prepared by the copolymerization of CO_2_ and BO at 25 °C for 12 h at a BO/cat/PPNCl mole ratio of 1000/1/1. The crude product (Figure 1A) and the purified product precipitated in ethanol (Figure 1B) were characterized by ^1^H NMR. The chemical shifts at 4.9 ppm and 4.2 ppm corresponded to the methine and methylene protons next to the carbonate linkage in the polycarbonate (Figure 1A). In addition, the methylene signals corresponding to the five-membered cyclic carbonate were observed at 4.5 ppm and 4.3 ppm. The peaks at 2.4 ppm, 2.7 ppm, and 2.8 ppm corresponded to the methylene and methine proton peaks of unreacted BO. In the ^1^H NMR spectra of the purified product, peaks corresponding to cyclic carbonate (h, g) and the raw material (e, f) disappeared completely, indicative of the synthesis of a pure polycarbonate (Figure 1B).

Copolymerization was considerably affected by the reaction temperature, reaction time, catalyst concentration, and CO_2_ pressure [37]. First, the effect of the reaction time on polymerization was examined. A series of polymerization reactions with different reaction times were conducted at 25 °C with a BO/cat/PPNCl feed mole ratio of 1000:1:1 (Table 1, entries 1–6). As expected, the conversion of the monomer increased gradually with time from 66.2% to 95.2%. Further studies revealed that the selectivity of the polymerized products decreased to 77.6% with increasing reaction time to 24 h, accompanied by a decrease in molecular weight to 40 kg mol^−1^. In general, it has been hypothesized that the metal-catalyzed copolymerization of CO_2_ and epoxides involves a coordination–insertion mechanism [38,39,40]. An assumption similar to that described above has also been proposed for binary SalenCo(III)X/nucleophilic co-catalyst systems, where two reaction routes are involved in the formation of cyclic carbonates: back-biting of the metal-bound carboxylate or the depolymerization of the propagating polycarbonates chain (unzipping) [41]. In the initial stage of the reaction, polycarbonates were formed selectively by the careful selection of the co-catalyst and reaction temperature. The prolonged reaction caused an increase in system viscosity and a decrease in mass and heat transfer, enabling the unzipping of the polymer. This result indicates that the selectivity of the polymerized product decreases with increasing reaction time, mainly due to the depolymerization of the resultant linear polycarbonate with a completely alternating arrangement, which produces more thermodynamically stable cyclic carbonate.

In addition, the effect of the catalyst concentration on the copolymerization of CO_2_ with BO at a reaction time of 12 h was evaluated (Table 1). With the decrease in the mole ratio to 500:1:1 (Table 1, entry 7 vs. 3), the BO conversion was significantly improved from 86.6% to >99%, and the selectivity of the polymerization product was also up to 99%. With the further reduction of the catalyst dosage with a BO/cat/PPNCl feed mole ratio of 2000:1:1, conversion of 67.8% with a TOF value of 113 h^−1^ was obtained (Table 1, entry 8), but the molecular weight was only 15.0 kg mol^−1^. When the mole ratio increased from 5000:1:1 and 10,000:1:1 (Table 1, entries 9 and 10), the purified polymer was barely obtained by the dissolution/precipitation process due to its low molecular weight. The ^1^H NMR spectrum also showed a decrease in conversion to 22.4% and 7.5%.

For the binary systems of SalenCoCl and PPNCl, the polymerization selectivity is highly sensitive to the reaction temperature. As can be observed from Table 1, the polymer selectivity was reduced to 46.9% at a reaction temperature of 40 °C (Table 1, entry 11). The further increase in the temperature decreased the selectivity to 39.7% (Table 1, entry 12). At 80 °C (Table 1, entry 13), the pressure of the reaction system dropped rapidly and then ceased dropping after a short time (e.g., 1 h). Accordingly, the reaction system reacted extremely rapidly at high temperatures, albeit with extremely poor selectivity. This result was consistent with the previously proposed mechanism: increasing the temperature led to a higher tendency for the reaction to produce a more thermodynamically stable cyclic carbonate. This result may be related to the fact that the counter cation is not linked intramolecularly to the cobalt complex. Therefore, the counter cation and dissociated anion can be far from the cobalt center, particularly at high reaction temperatures and/or in a highly diluted solution [42].

In addition, the effect of pressure on the reaction process was evaluated. After 12 h of reaction, the molecular weight of the polycarbonate gradually increased and then decreased at CO_2_ pressures of 1 MPa, 2 MPa, 3 MPa, and 4 MPa (Table 1, entries 14, 15, 3, 16, respectively). A molecular weight of 41.7 kg mol^−1^ was achieved at 3 MPa, and the molecular weight distribution did not change significantly under these conditions. Another effect of the pressure on polymerization is that, at low CO_2_ pressures, the carbonate unit content of PBC decreases, indicating that a small amount of polyether is produced, which is consistent with that reported in previous studies [43].

### 3.2. Terpolymerization of BO/CO_2_ and Other Epoxides

**Figure 2:**
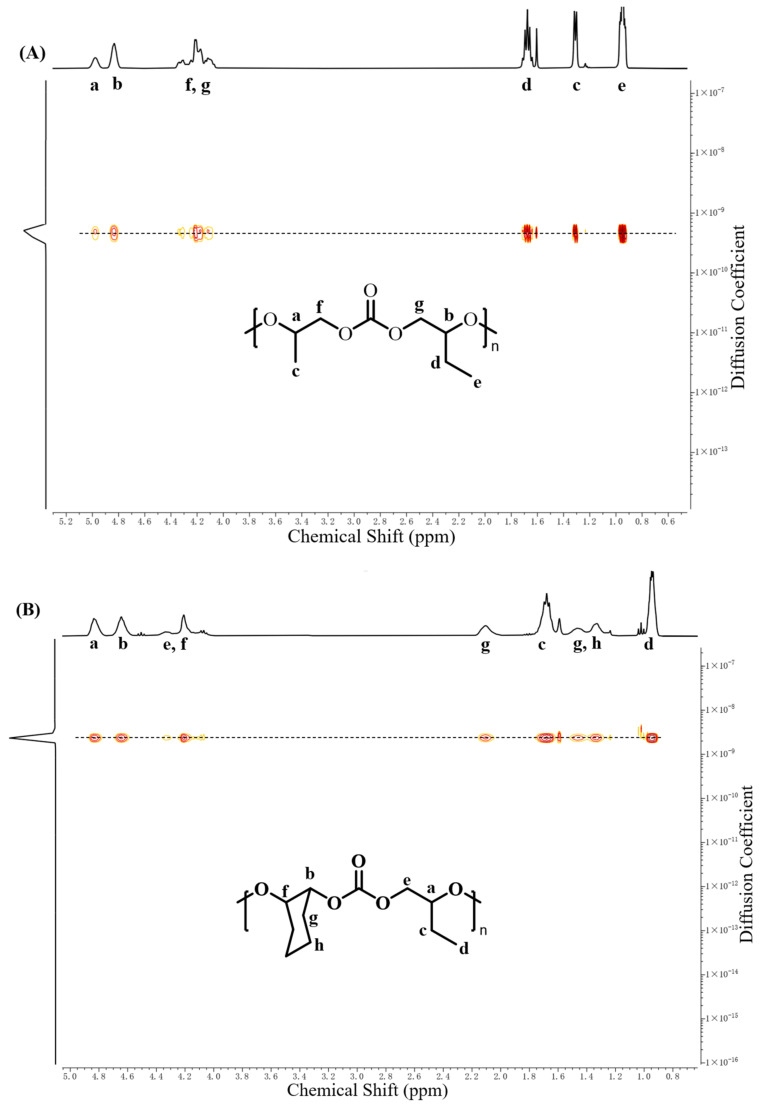
DOSY spectra of (**A**) PBC-co-PPC and (**B**) PBC-co-PCHC.

One of the advantages of epoxide/CO_2_ copolymerization is that polymer properties can be modified not only by varying the monomer but also by copolymerizing with another epoxide. It should be noted that although CO_2_/PO and CO_2_/CHO copolymers have been extensively studied, the lower glass transition temperature of PPC, the high brittleness of PCHC, and other inherent defects have seriously limited their application. The research on adjusting the T_g_ of polymers and improving their mechanical properties by introducing a third monomer has received much attention. The main difficulty in terpolymerization is the difference in reactivity between monomers, making it difficult to achieve effective control over the composition and structure of the polymer. Table 2 summarizes the results of the terpolymerization of BO/PO/CO_2_ and BO/CHO/CO_2_ catalyzed by the (R,R)-SalenCoCl and PPNCl binary system. To further clarify the terpolymer structure, and to exclude the possibility of the mechanical mixture of the two polymers (i.e., PBC and PPC), DOSY NMR analysis of the samples was conducted. The DOSY method is a form of two-dimensional nuclear magnetic resonance that is used to measure the diffusion coefficients of molecules. It is more suitable for distinguishing a mixture of two or three components with different self-diffusion factors. As shown in Figure 2, the DOSY spectra revealed only a single signal with a low diffusion coefficient, corresponding to the ^1^H NMR signals of PBC-co-PPC (Table 2, entry 18) and PBC-co-PCHC (Table 2, entry 21).

At a BO to PO feed ratio of 9:1 (Table 2, entry 17), the ratio of the PBC to PPC linkage in the terpolymer was 1:0.47. The polymer selectivity was ~96.0%, and the molecular weight was 58.8 kg mol^−1^. With the increase in the PO feed ratio, terpolymerization was more likely to occur, leading to an increase in the PPC linkage of the polymer chains. In addition, the selectivity of the polymer was decreased, its TOF value was not significantly altered, and its molecular weight was increased. The PBC to PPC linkage ratio in the terpolymer was 1:1.41 with a BO feed ratio of 5:5 (Table 2, entry 19). Accordingly, the polymer selectivity was decreased to 91.1%, and the molecular weight was 68.6 kg mol^−1^. In addition, the terpolymerization of CO_2_, BO, and CHO was investigated. At a BO to CHO feed ratio of 9:1 (Table 2, entry 20), the PBC to PCHC linkage of the polymer was 1:0.37, while the selectivity of the polymer was 97.1%, and the molecular weight was 32.2 kg mol^−1^. The molecular weight of the product increased first and then decreased with the increasing CHO feed ratio; at a BO to CHO feed ratio of 5:5, the molecular weight reached 48.4 kg mol^−1^ (Table 2, entry 22).

### 3.3. Thermal Analysis of PBC, PBC-co-PCHC, and PBC-co-PPC

**Figure 3:**
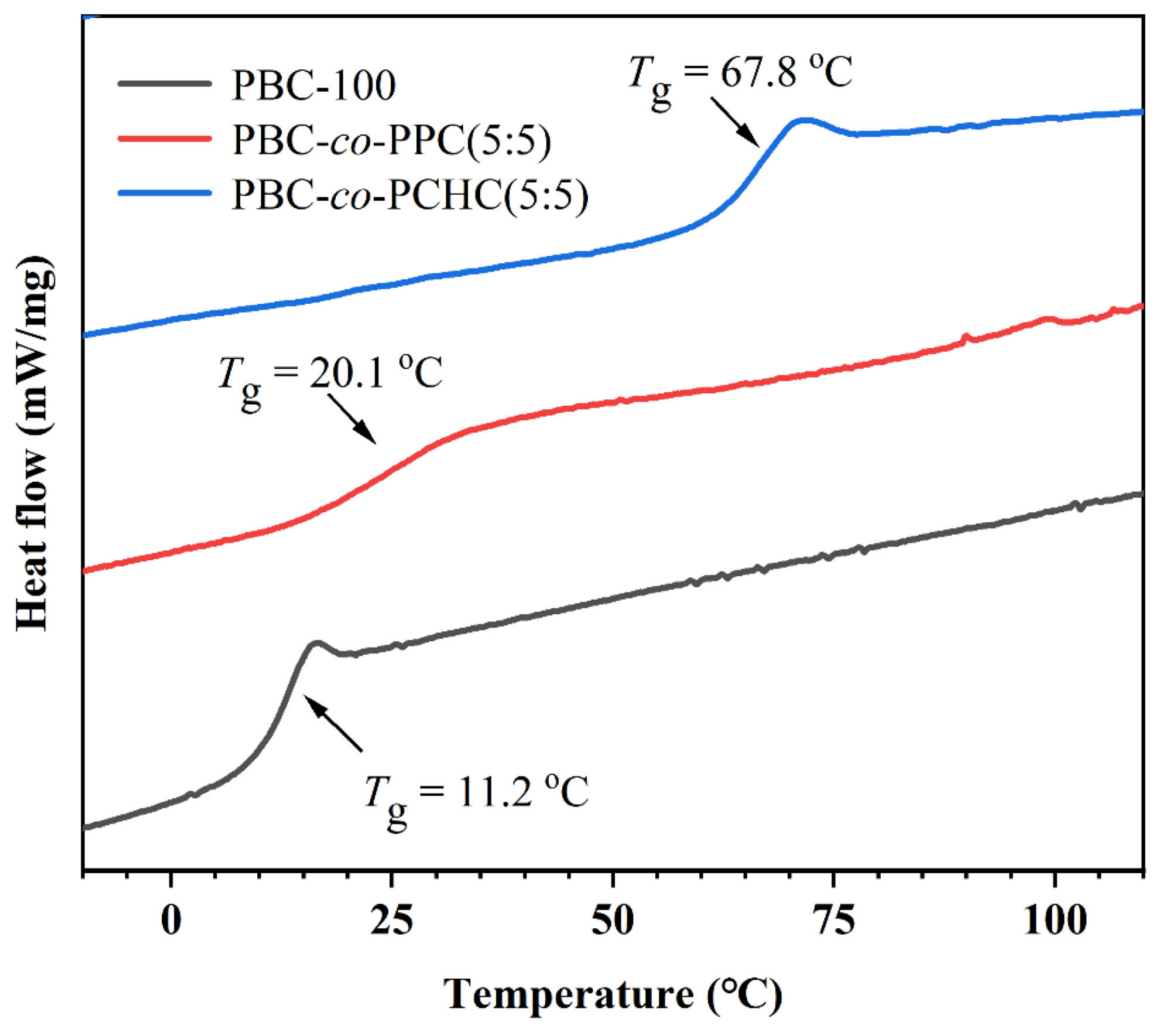
DSC analysis of the obtained polycarbonates.

DSC was employed to measure the T_g_ of the resultant copolymer and terpolymer. As shown in Figure 3, the T_g_ of PBC was 11.2 °C (Table 2, entry 3). All of the terpolymers in Table 2 with different molar ratios of BO (PO or CHO) exhibited a single T_g_ (Figure 3). At a BO:PO feed ratio of 9:1, T_g_ was 14.3 °C. When the PO feed ratio was increased to 1:1, the T_g_ of the resulting polymer increased to 20.1 °C. This result was related to the increase in the PO content of the polymer, which stiffened the chain segments; thus, their glass transition temperatures increased. In general, however, the introduction of BO further lowers the glass transition temperature of PPC, which is unfavorable for its use as a raw material for disposable film packaging due to its low glass transition temperature. In contrast, it is more practical to introduce BO into the copolymerization of CHO and CO_2_, resulting in the modulation of the material’s mechanical properties and its glass transition temperature. Therefore, the terpolymerization of BO, CHO, and CO_2_ was also investigated with a further increase in the T_g_ of the polymer. At a BO:CHO ratio of 9:1, a polymer with a molecular weight of 32.2 kg mol^−1^ and a T_g_ of 32.4 °C was produced. With the increase in the CHO content of the feed ratio, the chain rigidity and T_g_ increased, and at a feed ratio of 1:1, T_g_ reached 67.8 °C. This result was attributed to the presence of longer side chains of BO, resulting in stronger plasticizing effects and regioirregular microstructures. With the addition of PO/CHO, the otherwise flexible PBC chain segments slowly hardened, making the polycarbonate more rigid and increasing its T_g_.

**Figure 4:**
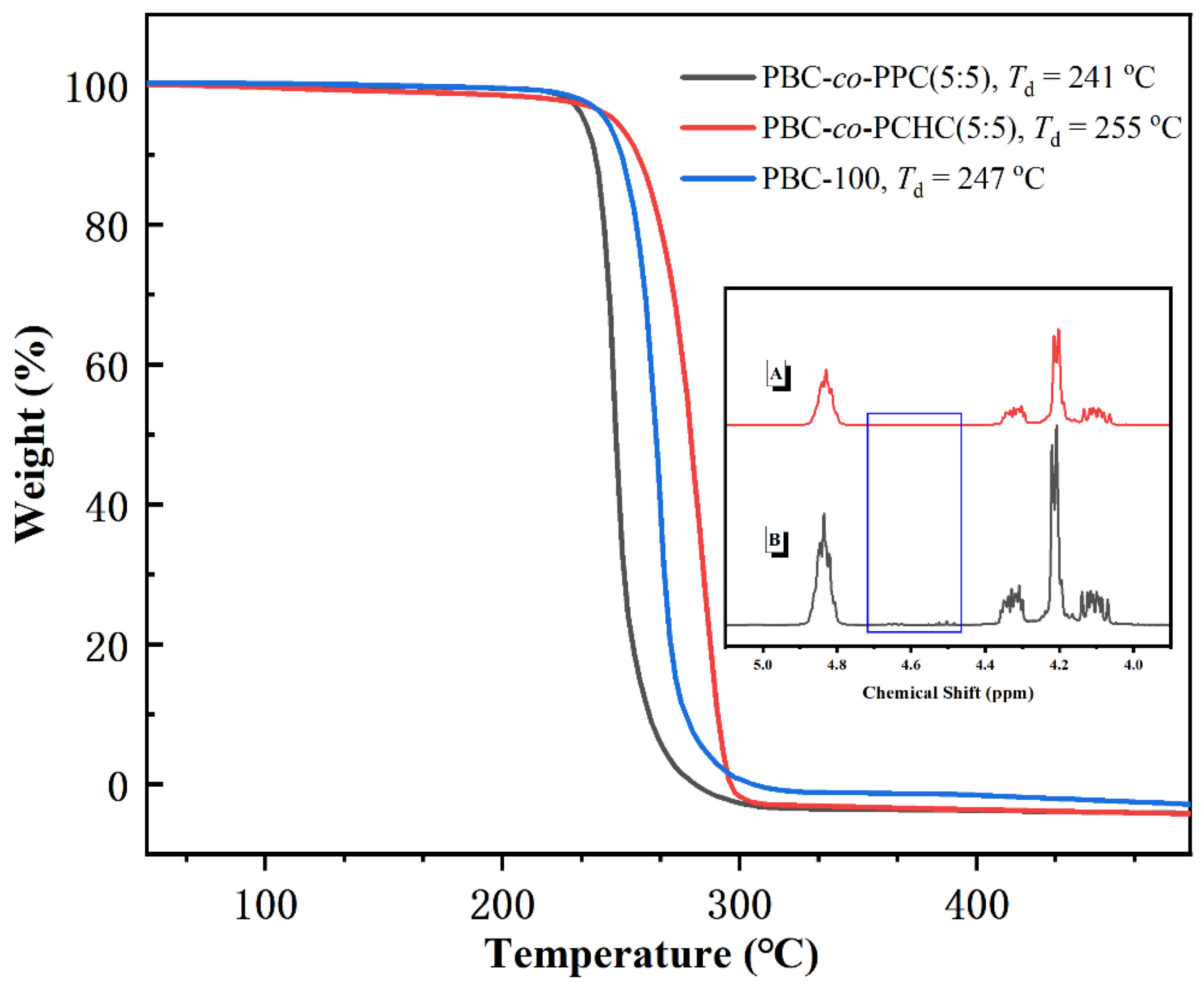
TGA curves of the obtained polycarbonates and ^1^H NMR of pure PBC (**A**) after heating for 10 min (**B**).

In addition, TGA was employed to analyze the thermal stability of the obtained polycarbonates. As shown in Figure 4, the T_d_ of PBC-co-PPC at 5 wt% loss was 241 °C, which was less than those of PBC at 247 °C and PBC-co-PCHC at 255 °C.

It is important to note that in some systems, the decomposition of polymers (unzipping) may occur at very low temperatures due to the presence of the catalyst, but the resultant cPC cannot be volatilized at such low temperatures, making the change invisible on the TGA curve. To determine whether this is the case for our system, a control experiment was performed. The ^1^H NMR spectra before and after holding the polymer at a constant temperature of 240 °C for 10 min (inset in Figure 4) revealed low polymer degradation (the heated spectra revealed insignificant peaks of cyclic carbonate at 4.5 ppm and 4.3 ppm), but this change was almost negligible (the percentage of integrated area was greater than 99%). The results indicate that the unzipping reaction of the polycarbonate at low temperatures is insignificant during the extremely short TGA test.

### 3.4. Contact Angle Measurements

**Figure 5:**
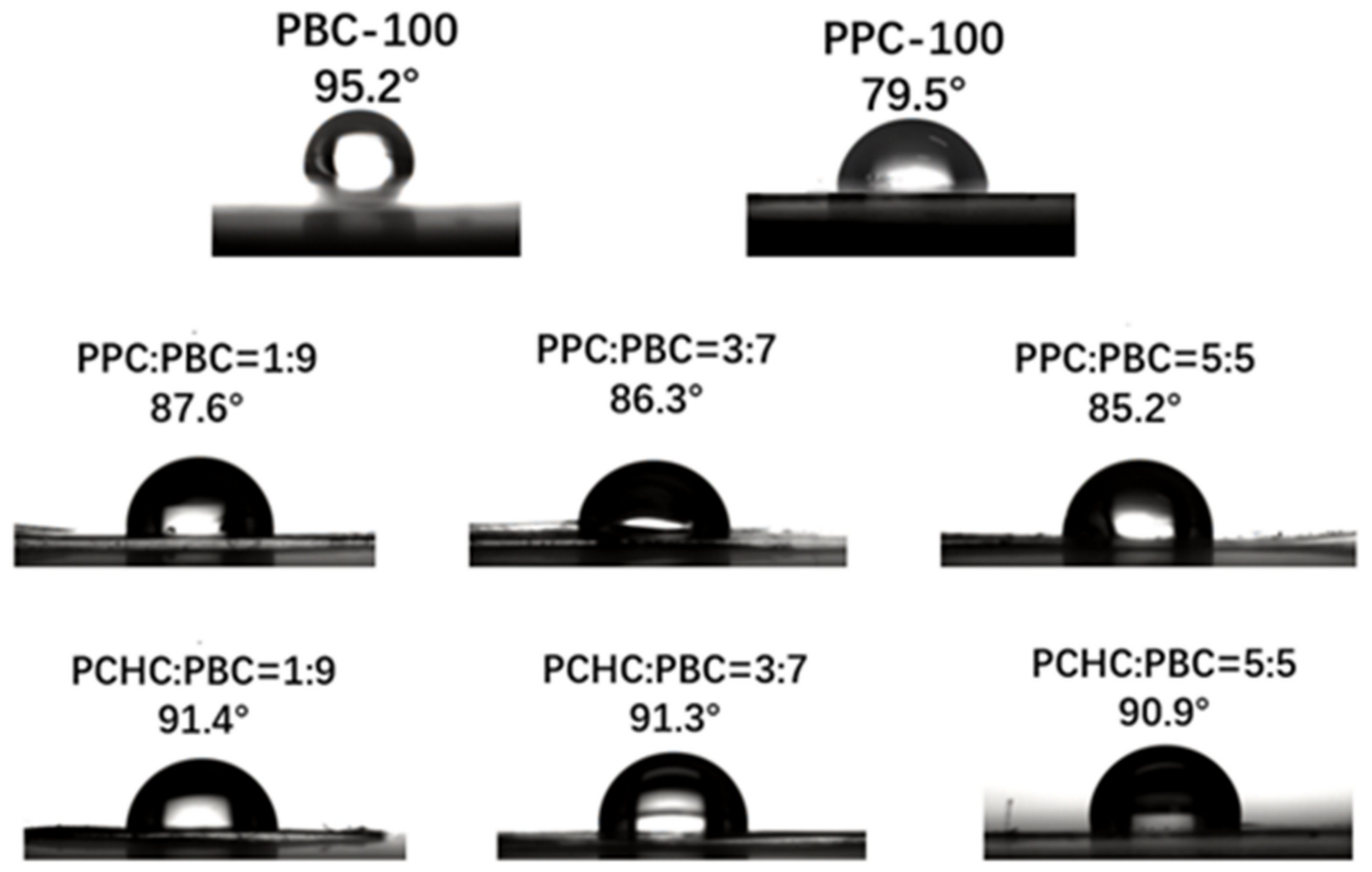
Water contact angles of CO_2_-based polycarbonates.

The hydrophobic and hydrophilic performance of polymers is well known to be an important factor for their biodegradability. Hydrophilicity promotes enzymatic degradation due to the presence of abundant surface water molecules. Therefore, an appropriate balance of hydrophobic and hydrophilic characteristics provides an opportunity to regulate the degradation rate of polymers [44,45]. Polybutylene oxide is more hydrophobic than polypropylene oxide. Thus, the introduction of the BO unit into PPC may enhance the hydrophobicity of the polymer. Thus far, only a few studies have investigated the surface properties of CO_2_-based polycarbonates [46,47]. Herein, the contact angles of PBC and terpolymers were measured. The contact angles of pure PBC and PPC were 95.2° and 79.5°, respectively. The replacement of the methyl group in PO with an ethyl or cyclohexane ring (in BO and CHO) resulted in the enhanced hydrophobicity of the polymer, i.e., the contact angle increased with the increase in the PBC content (Figure 5). The same result was observed for the terpolymers of CHO, BO, and CO_2_: with the increase in PBC content, the contact angle increased. Thus, this terpolymerization method offers a modification method for the surface characteristics of CO_2_-based polymers. The effect of surface hydrophilicity on the degradation rate is still being investigated.

## 4. Conclusions

In summary, the alternating copolymerization of CO_2_ with BO and the terpolymerization of CO_2_, BO, and PO or CHO were successfully realized by using a binary SalenCoCl catalyst. In addition, the effects of the polymerization reaction conditions and copolymerization monomer composition on the polymer structure and properties were investigated. The thermal properties of polycarbonates such as T_g_ and T_d_ can be easily adjusted by alternating the epoxide feed ratio. In addition to modulating the thermal properties, a third monomer, such as PO or CHO, also can alter the hydrophobicity of CO_2_/BO polycarbonates, providing an opportunity to modulate the degradation rates.

## Figures and Tables

**Table 1:** Copolymerization of CO_2_ and BO.

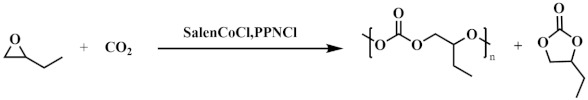								
Entry	cat:cocat:BO	Time (h)	Conv. (%) ^2^	CU (%) ^3^	Selectivity (%) ^4^	TOF (h^−1^) ^5^	M_n_ (kg mol^−1^) ^6^	Đ ^6^
1 ^1^	1:1:1000	4	66.2	>99	>99	166	19.5	1.31
2 ^1^	1:1:1000	8	75.6	>99	>99	95	28.0	1.34
3 ^1^	1:1:1000	12	86.6	>99	98.1	72	41.6	1.27
4 ^1^	1:1:1000	16	92.6	>99	88.5	57	38.2	1.28
5 ^1^	1:1:1000	20	93.7	>99	80.0	46	36.9	1.30
6 ^1^	1:1:1000	24	95.2	>99	77.6	40	21.7	1.35
7 ^1^	1:1:500	12	>99	>99	>99	42	36.3	1.26
8 ^1^	1:1:2000	12	67.8	>99	>99	113	15.0	1.27
9 ^1^	1:1:5000	12	22.4	>99	>99	93	/	/
10 ^1^	1:1:10,000	12	7.5	>99	94.3	63	/	/
11 ^7^	1:1:1000	12	93.3	>99	46.9	78	31.1	1.12
12 ^8^	1:1:1000	12	>99	>99	39.7	83	24.7	1.46
13 ^9^	1:1:1000	12	83.6	>99	12.5	70	20.5	1.20
14 ^10^	1:1:1000	12	70.9	98.0	>99	59	26.0	1.28
15 ^11^	1:1:1000	12	72.5	>99	>99	60	31.0	1.27
16 ^12^	1:1:1000	12	84.7	>99	>99	70	31.1	1.30

^1^ The copolymerization was carried out at 25 °C and 3 MPa unless noted otherwise. ^2^ Conversion of BO determined by ^1^H NMR analysis of the crude reaction mixture. ^3^ The content of carbonate unit. ^4^ Percentage of polymer formed vs. cyclic carbonate as determined by 1H NMR. ^5^ TOF = turnover frequency, mol of epoxide consumed × mol of SalenCoCl^−1^ × h^−1^. ^6^ Determined by GPC in THF, at 30 °C, calibrated with polystyrene standards. ^7^ The reaction temperature was 40 °C. ^8^ The reaction temperature was 60 °C. ^9^ The reaction temperature was 80 °C. ^10^ The reaction pressure was 1 MPa. ^11^ The reaction pressure was 2 MPa. ^12^ The reaction pressure was 4 MPa.

**Table 2:** Terpolymerization of CO_2_, BO, and other epoxides.

Entry	Epoxide	BO:epo ^1^	PBC:PPC/PCHC	Selectivity (%)	M_n_ (kg mol^−1^) ^2^	Đ ^2^	T_g_ (°C) ^3^
3	\	\	\	98.1	41.6	1.27	11.2
17	PO	9:1	1:0.47	95.6	58.8	1.12	14.3
18	PO	7:3	1:1.15	95.2	62.7	1.08	15.9
19	PO	5:5	1:1.41	91.1	68.6	1.07	20.1
20	CHO	9:1	1:0.37	97.1	32.2	1.14	32.4
21	CHO	7:3	1:1.02	98.0	50.7	1.12	42.6
22	CHO	5:5	1:2.22	97.1	48.4	1.11	67.8

^1^ Mole ratio of epoxide, [epoxide]:SalenCoCl:PPNCl = 1000:1:1. ^2^ Determined by GPC in THF, at 30 °C, calibrated with polystyrene standards. ^3^ Determined by DSC.

## Data Availability

Not applicable.
